# The prognostic value of the ratio of neutrophils to lymphocytes before and after intensity modulated radiotherapy for patients with nasopharyngeal carcinoma

**DOI:** 10.1097/MD.0000000000018545

**Published:** 2020-01-10

**Authors:** Jing Liu, Changwu Wei, Haijun Tang, Yun Liu, Wenqi Liu, Chengsen Lin

**Affiliations:** a Department of Radiation Oncology, The Second Affiliated Hospital of Guangxi Medical University; b Department of Orthopaedics, The First Affiliated Hospital of Guangxi Medical University; c Department of Orthopaedics, Minzu Affiliated Hospital of Guangxi Medical University, Nanning, Guangxi, China.

**Keywords:** nasopharyngeal carcinoma, neutrophil-to-lymphocyte ratio, platelet-to-lymphocyte ratio, prognosis, radiotherapy

## Abstract

This study aimed to determine the impact of the neutrophil-to-lymphocyte ratio (NLR) and the platelet-to-lymphocyte ratio (PLR) on the prognosis of nasopharyngeal carcinoma (NPC) before and after intensity modulated radiotherapy (IMRT).

Pre/post-treatment and changes in inflammatory biomarker levels of 207 patients who were diagnosed with NPC and received IMRT between January 2012 and December 2014 were analyzed, and the cellular biomarker analyses were from patient blood. ROC (receiver operating characteristic) analysis was used to decide the optimal cutoff values of NLR and changes in NLR (ΔNLR) and PLR (ΔPLR). The Kaplan–Meier and logarithmic rank methods were used to compare overall survival times between groups. Univariate analysis was used to investigate the effects of age, gender, histology, Karnofsky performance score (KPS), TNM stage, clinical stage, course of disease and lymphocyte, neutrophil and platelet counts as well as alkaline phosphatase (ALP) levels on the prognosis of NPC. The independent predictors of OS were determined by Cox multivariate regression analysis.

The optimal cut-off values of NLR, PLR, ΔNLR and ΔPLR were 2.49, 155.82, 1.80, and 100.00, respectively. These were used to classify patients into high (NLR > 2.49) and low NLR groups (NLR < 2.49); high (PLR>155.82) and low (PLR < 155.82) PLR groups; high (ΔNLR>1.80) and low ΔNLR groups (ΔNLR < 1.80); high (ΔPLR > 100.00) and low ΔPLR groups (ΔPLR < 100.00). TNM stage, clinical stage and ALP levels were highly correlated with high NLR and PLR. Cox multivariate regression analysis suggested that the ΔNLR (HR = 2.89, 95% CI: 1.33∼2.78) was independent of the characteristics for NPC.

As a novel inflammatory index, ΔNLR appears to have some predictive power for the prognosis of patients with NPC.

## 1 Introduction

Nasopharyngeal carcinoma (NPC) is an epithelial cancer that originates from the mucous layer of nasopharynx, usually within the lateral nasopharyngeal recess.^[1]^ There are large regional differences in the incidence of nasopharyngeal cancer, which is relatively low in western countries but as high as 25/100,000 in the south of China. Therefore, nasopharyngeal cancer can be considered to be an endemic disease in this area of China.^[2]^ NPC is found in the nasopharynx, which is located behind the nose and above the back of the throat, thus making it inaccessible to radical surgery. However, as NPC originates from the epithelial cells of the nasopharyngeal mucosa, most of its histological types are non-keratinizing undifferentiated carcinoma, and it is highly sensitive to radiation. Therefore, radiotherapy has become the main approach for treating this disease.^[3]^

Radiotherapy, as a physiotherapy method of local ablation, can induce double-stranded DNA damage of cancer cells. With high-energy radiation, single-stranded damage, error repair and chromosomal aberrations, can achieve better curative effects in NPC.^[4–8]^ However, in clinical practice, there is still a risk of disease recurrence and distant metastases after radiotherapy. More importantly, among patients with NPC with the same TNM stage, there is considerable heterogeneity in achieved clinical results, but the reasons for which have not been fully identified.^[9]^

The interaction between inflammatory and tumor cells can change the tumor tissue homeostasis and can construct a different tumor microenvironment (TME).^[10]^ A large number of inflammatory cells interact with inflammatory cytokines in the TME, promoting tumor progression through mechanisms such as pro-inflammatory immune-editing and immune escape, and tumor cells secrete cytokines that can also change the number and phenotype of inflammatory cells.^[4]^ Among them, the increase in the number of neutrophils, platelets and lymphocytes have been found to be closely related to the poor prognosis of tumors.^[11]^ Therefore, through the number of inflammatory cells (such as platelets, neutrophils, lymphocytes and mononuclear cells), and the ratios between them, such as the ratio of neutrophils-to-lymphocytes ratio (NLR) and platelet-to-lymphocyte ratio (PLR) lymphocytes-to-monocytes ratio (LMR), have been shown to associated with the prognosis of a variety of malignant tumors.^[12–16]^ In the study of NPC, high NLR and PLR have been demonstrated to be associated with a bad prognosis. Other studies have revealed that high NLR^[17]^ and LMR^[18,19]^ can act as a biomarker for metastatic NPC. As a result, inflammatory markers are currently viewed as prognostic indicators of NPC.

However, recent studies have shown that in addition to the direct destruction of cancer cells by radiation during radiotherapy, it can also activate the immune response and affect the changes in the level of inflammatory cells in the TME, resulting in changes in the number and phenotype of neutrophil lymphocytes.^[20,21]^ Currently, the reported literature emphasizes the predictive value of NLR and PLR levels in cancer prognosis before treatment, while ignoring the effect of radiotherapy on TME inflammatory cells. It is unclear about the predictive value of these changes in prognosis. Consequently, the aim of this retrospective study was to observe the changes in the ratios of NLR and PLR before and after radiotherapy, as well as the prognostic effect and clinical significance of these changes in NPC.

## 2 Methods

### 2.1 Patients

We retrospectively reviewed the clinical data of patients diagnosed with NPC at the First and Second Affiliated Hospitals of Guangxi Medical University between January 2012 and December 2014.

The inclusion criteria were as follows:

(1) NPC was confirmed by histopathological and imaging findings based on the American Joint Committee on Cancer, 7th edition, TNM classification and staging system;
(2) all patients provided a detailed medical history and inflammatory markers were measured before and after anti-tumor therapy, which was either radiotherapy or chemotherapy;
(3) patients had no chronic diseases such as diabetes or COPD;
(4) patients had no blood and infectious diseases or fever;
(5) all patients received their treatment in our hospitals.

This study received the support of the ethics committee of the First and Second Affiliated Hospitals of Guangxi Medical University (Guangxi, China).

### 2.2 Clinical parameters

Different types of information were acquired from the patients’ medical records including imaging data, clinical information as well as laboratory test results. Clinical data from each patient were collected the day before chemo-radiotherapy or within 7 days after concurrent therapy began including: age, sex, pathological type, Karnofsky performance score (KPS) as well as the radiotherapy technique and dose used. Laboratory tests include: blood counts (lymphocyte, neutrophil, monocyte, platelet and white blood cell counts) as well as percentages of neutrophils, lymphocytes and platelets. NLR was determined by dividing the absolute of neutrophils count by the absolute count of lymphocytes. PLR was determined by dividing absolute platelets count by the absolute count of lymphocytes. The delta change in inflammatory biomarkers was calculated by subtracting the levels of inflammatory biomarkers before intensity modulated radiotherapy (IMRT) to those after IMRT.

### 2.3 Treatment

During the study period, our treatment strategy was guided mainly by the National Cancer Integrated Network Guidelines (NCCN). The cases with early-stage (I stage) of the disease were treated with RT alone, while loco-regionally advanced-stage illness (II-IV stage) was treated with concurrent chemo-radiotherapy. Three chemotherapy regimens were administrated every 21 days, a total of 2 to 3 cycles of CCRT were applied during RT:

(1) TP regimen: paclitaxel (175 mg/m2/d over 3 hours) with added cisplatin (25 mg/m2/d1–3);
(2) PF regimen: cisplatin (25 mg/ m2/d1–3) with added 5-fluorouracil (500 mg/ m2/d1–5);
(3) TPF regimen: paclitaxel (135 mg/ m2/d1 over 3 hours) with added cisplatin (25 mg/ m2/d1–3) and 5-fluorouracil (800 mg/ m2/d1–5).

The general dose of IMRT for NPC was 66 to 72 Gy, for cervical lymph node positive cases it was 60 to 70 Gy, for high-risk areas it was 60 Gy and for prophylactic irradiation areas it was 50 to 54 Gy.

### 2.4 Outcome and follow-up

After treatment, the patients received a phone call, letter or outpatient interview every 3 months during the first 2 years after which this changed to every 6 months for up to a 5-year period. After that, they were followed up once a year. All patients continued to be followed up until death from NPC or July 31st 2019, whichever came first. Routine follow-up items included a physical examination, whole blood count, blood biochemical tumor markers, CT scan and b- ultrasound examination. From the beginning of treatment to the day of death, all treatment and follow-up records of these patients were documented.

We selected the primary endpoint as overall survival (OS) which was calculated from initial treatment to disease-specific death. For recurrence, we recorded the latencies to the first remote or locoregional relapse, respectively and the CT and MRI was used to confirm whether there was recurrence.

### 2.5 Statistical analysis

The optimal cutoff values for all markers were assessed by time-dependent ROC curve and area under curve (AUC) analyses. The standard error of the mean (SEM) was used to represent the measurement data subject to normal distribution, and the median (quartile) was used to represent the measurement data subject to normal distribution. The optimal cut-off values of NLR, PLR, ΔNLR, ΔPLR were determined by ROC analysis. *T*-tests were carried out for 2 sets of data which were normally distributed, and the chi-square test was used to compare data which were not normally distributed. Survival curves were created by the Kaplan–Meier methodology and compared with the help of the log-rank test. The results of Cox multivariate regression analysis modeling were expressed as hazard ratios (HRs) and were correlated at 95% confidence intervals (CIs). In addition, variables with *P* < .05 in univariate analysis were included in multivariate analysis. *P* < .05 was considered to be statistically significant. SPSS 20.0 (SPSS, Inc. Chicago, IL) was used to for statistical evaluations.

## 3 Results

The clinic-pathological features of the patients were shown in Table 1. Among the 207 patients having NPC, there were 112 males (54.11%) and 95 females (45.89%). The median age was 45 years. Based on the AJCC grading standards, 109 patients (52.76%) were at stages I and II, and 98 patients (47.24%) were at stages III and IV. 106 patients (51.21%) underwent concurrent chemo-radiotherapy while 101 patients (48.89%) underwent RT alone.

**Table 1:**
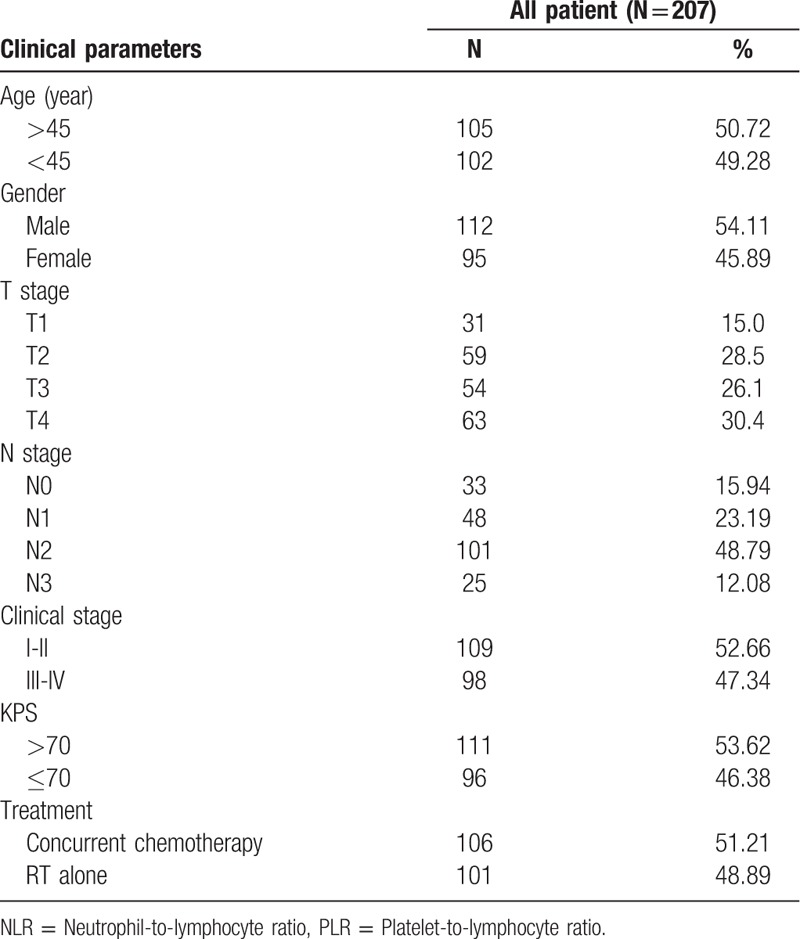
Association of the patients’ clinical parameters with NLR and PLR.

### 3.1 The optimal cutoff values of NLR, PLR, ΔNLR, ΔPLR

**Figure 1:**
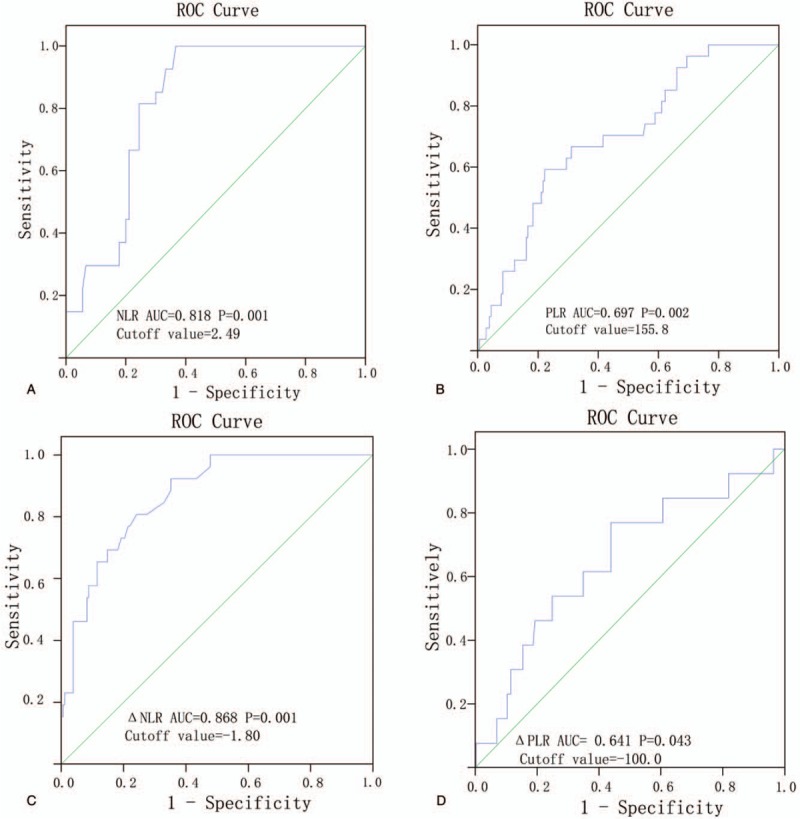
ROC curve for OS with respect to NLR, PLR, ΔNLR, and ΔPLR in patients with nasopharyngeal carcinoma. Cutoff value of NLR, 2.49 (A); Cutoff value of PLR (B), 155.82; Cutoff value of ΔNLR (C), –1.80; Cutoff value of ΔPLR (D), –100.00. NLR = Neutrophil-to-lymphocyte ratio, OS = Overall survival, PLR = Platelet-to-lymphocyte ratio, ROC = receiver operating characteristic.

The death outcome of patients with NPC was used as the endpoint, and Figure 1 demonstrated the results of ROC analysis of NLR, PLR, ΔNLR, and ΔPLR. The analysis demonstrated that the AUC corresponding to NLR, PLR, ΔNLR, and ΔPLR were 0.818 (*P* = .002), 0.697 (*P* = .014), 0.868 (*P* = .001) and 0.641 (*P* = .021), respectively (Fig. 1). At the highest Youden index, the indicator threshold was the best one for distinguishing the predicted target variables. Thus, the best cut-off values of NLR, PLR, ΔNLR, and ΔPLR were 2.49 (sensitivity, 92.86%; specificity, 36.67%), 155.82 (sensitivity, 66.67%; specificity, 31.11%), 1.80 (sensitivity, 92.31%; specificity, 35.16%) and 100.00 (sensitivity, 76.92%; specificity, 45.05%), respectively. The Youden indices of NLR, PLR, ΔNLR along with ΔPLR were 0.633, 0.356, 0.571, and 0.319, respectively. As a result, based on the optimal cut-off values of NLR, ΔNLR, PLR, and ΔPLR, the patients were divided into a high (Δ NLR > 1.80) and low Δ NLR groups (Δ NLR < 1.80), a high (PLR > 155.8) and low PLR groups (PLR < 155.8), a high (Δ PLR > 100.0) and low Δ PLR groups (Δ PLR < 100.0), a high (NLR > 2.49) and low NLR groups (NLR < 2.49)

### 3.2 Prognosis condition of patients of the extremities after IMRT during follow-ups

The 207 patients with NPC were followed for a maximum of 82 months. The mean survival time for patients alive and dead was 33.7 (range, 10–61) months and 87.4 (range, 24–82). During the follow-up period, 15 patients were lost because we were unable to reach these patients or their families. In addition, recurrence was recorded in 8 patients. Factors influencing the postoperative prognosis of patients with NPC of the extremities. Regression univariate analysis was used to determine the factors that influenced the recurrence rate and this is shown in Table 2. N stage, clinical stage, NLR, PLR, ΔNLR as well as ΔPLR were found to be significant determinants of prognostic outcome. These factors were therefore included in the Cox multivariate regression analysis in order to carry out multivariate analysis together with the consideration of age and gender. The results of Table 2 demonstrated that ΔNLR were independent elements affecting the prognosis. Specifically, ΔNLR (HR = 2.89, 95% CI: 1.33–2.78) was a risk element for the recurrence of NPC after IMRT.

**Table 2:**
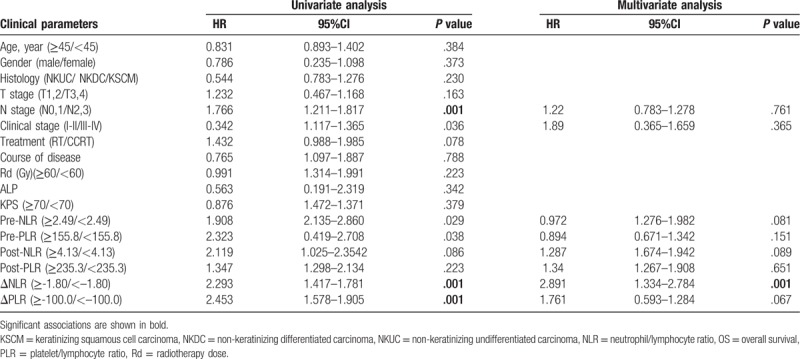
Univariate and multivariate survival analyses of OS in patients with inoperable NPC.

## 4 Discussion

In our study, we investigated 207 patients from two hospitals in Nanning, Guangxi. The results show the difference before and after IMRT. ΔNLR can be considered as a prognostic indicator for the OS of NPC. To our knowledge, this is the first report that NLR changes in patients with NPC before and after radiotherapy and chemotherapy are associated with clinical significance and prognosis. In addition, we found that there was a link between ΔNLR before and after treatment and OS rates in patients with NPC and that this was a useful prognostic indicator of the disease.

Rudolf Virchow^[22]^ was the first person to propose a possible connection between inflammation and cancer. In recent years, there has been clear evidence showing that inflammation plays a vital role in tumorigenesis.^[23–25]^ In addition, some latent molecular mechanisms have been clarified.^[26]^ The progression of cancer is determined not only by the malignant behavior of tumors, but also by the systemic inflammatory response expressed by several immune-related cells.^[24]^ The interaction of tumor cells with the extracellular matrix and surrounding stromal cells promotes the TME.^[26,27]^ Fang and Declerck^[27]^ showed that cancer cells and their microenvironment promote proliferation, evade growth inhibitors, resist apoptosis, induce angiogenesis, as well as activate invasion and metastasis.^[24]^

Neutrophilic platelets and monocytes are considered to be important regulators of tumor invasion and metastasis.^[22,28]^ On the contrary, lymphocytes play an important role in protecting immunity by inhibiting the proliferation and migration of tumor cells.^[29,30]^ Kinoshita et al^[13]^ reported that low absolute lymphocyte counts were correlated with longer survival times (*P* = .050). In addition, Gooden et al^[31]^ described that circulating neutrophils may inhibit lymphocytosis, thereby eliminating host defense and immune surveillance, leading to carcinogenesis. In summary, we suspected that NLR may affect survival outcomes in NPC due to the combination of neutrophils and lymphocyte counts.

As expected, in our study, we found that there was an explicit connection between NLR before and after IMRT and OS, and the difference between NLR before and after IMRT was also related to OS for patients with NPC. Therefore, we analyzed the correlation between PLR and OS expression before and after IMRT, but, contrary to our expectation, found no significant correlation between NLR and survival rate in patients with NPC before radiotherapy. A possible reason for this is that cancer treatment itself can induce a strong tumor-related inflammatory response. Radiation and chemotherapy result in a large number of necrosis and death of cancer cells as well as the surrounding tissues, which in turn cause an inflammatory response similar to the wound healing response.^[27]^ Therefore, chemo-radiotherapy can further affect the TME. Therefore, we further analyzed the changes of inflammatory indicators before and after treatment. However, in univariate analysis, pre-NLR, pre-PLR, post-NLR and post-PLR were all correlated with OS, while no such correlation was found in the multivariate analysis, which was contrary to the study described by Li et al.^[32]^

**Figure 2:**
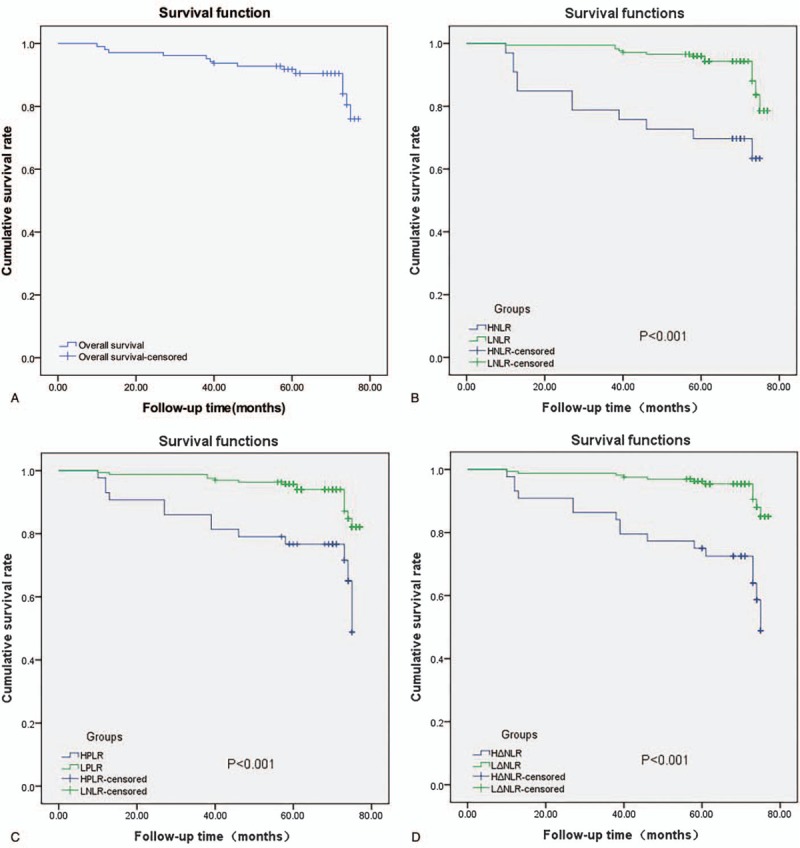
Overall survival curve of patients with nasopharyngeal carcinoma in different NLR, PLR, and ΔNLR. The death rate of tumors in patients in low RLR group was lower compared to that in high NLR group (B). The death rate of tumors in patients of low PLR group was lower than those in high PLR group (C). The death rate of tumors in patients of low ΔNLR group was lower than those in high ΔNLR group (D). NLR = Neutrophil-to-lymphocyte ratio, PLR = Platelet-to-lymphocyte ratio.

In the 207 patients we studied, there were significant increases in NLR and PLR during treatment, thus emphasizing the damage to the immune system. However, ΔNLR was found to be superior to other indicators for predicting outcome of NPC. We showed that ΔNLR increased after radiotherapy, which was negatively correlated with the possibility of therapeutic response, and this finding is consistent with the previously reported fact that sustained high levels of neutrophils was associated with poor therapeutic effect and prognosis of NPC.^[33–35]^ Considering that the effect of radiation and chemotherapy together with the host antitumor immune responses could cause multiple factors effect, we speculated the evaluation ΔNLR between pretreatment and after treatment NLR would be a more accurate indicator of the disease. In addition, our study also demonstrated that patients having relatively small changes in inflammatory indexes after IMRT had a better prognosis (Fig. 2). In our study, in addition to inflammatory biomarkers, other clinicopathological characteristics were also independent indicators to judge the prognosis, which was consistent with other studies.^[36,37]^ We found that the worse the KPS, the shorter the OS and the later the N stage, the worse the OS had higher predictive effects.

The 207 patients with NPC were further divided into high (44 cases) and low ΔNLR groups (164 cases). We found that in high ΔNLR patients, the median survival (70 months) was higher than the low ΔNLR patients (62 months). This suggests that a dynamic assessment of inflammatory status may be a more accurate predictor of patient outcome. Hence, dynamic changes in NLR were correlated with survival and radiation response, and poor prognosis was observed as NLR values moved higher. Giuliani et al^[38]^ reported an independent correlation between NLR and OS before treatment, which our study did not confirm, possibly because our sample size was not large enough.

Given the preclinical evidence supporting the role of radiotherapy in inducing lymphocyte infiltration,^[39]^ relatively low post-treatment NLR may be secondary to an anti-tumor lymphocyte response stimulated by radiotherapy, thereby improving survival. On the contrary, if the tumor is promoting a state of immune tolerance, we speculated that radiotherapy to kill tumor cells will result in a reversal of the tumor induced immunosuppression ability. However, we found no correlation between NLR after post-treatment of the disease. This was also the case for hepatocellular carcinoma^[40]^ and in locally advanced rectal cancer.^[41]^ The significance of the increased NLR is unclear, and we hypothesize that it may represent a persistent inflammatory state after radiotherapy.

However, there are some limitations in this study. First of all, only a few patients were retrospectively studied. Secondly, inflammatory biomarkers may be influenced by infection and although patients having active infection were excluded from this study, we have no data on the presence of latent infection. Finally, patients having the same tumors did not receive the same treatment, which may affect the interpretation of the results. Therefore, larger studies are needed to confirm the relationship between inflammatory biomarkers and prognosis of patients with NPC.

## 5 Conclusions

The results of this study demonstrated that before and after radiotherapy, ΔNLR was independent risk element of NPC, can this can predict the prognosis of the disease. Since this is already an integral routine blood test, NLR has the advantages being a simple, convenient and economic indicator of NPC. In the future, randomized prospective studies with larger sample size are needed to determine the relationship between inflammatory markers and NPC, coupled with more extensive studies on the mechanisms involved in the disease.

## Acknowledgments

We would like to thank Dr. Dev Sooranna, Imperial College London, for editing the manuscript.

## Author contributions

**Conceptualization:** Jing Liu, Haijun Tang, Wenqi Liu.

**Data curation:** Jing Liu, Haijun Tang.

**Funding acquisition:** Chengsen Lin.

**Investigation:** Changwu Wei, Chengsen Lin.

**Methodology:** Wenqi Liu, Chengsen Lin.

**Project administration:** Changwu Wei, Haijun Tang, Wenqi Liu.

**Resources:** Changwu Wei, Yun Liu.

**Software:** Yun Liu.

**Supervision:** Wenqi Liu.

**Validation:** Yun Liu, Wenqi Liu.

**Writing – original draft:** Jing Liu.

**Writing – review & editing:** Jing Liu.
